# Nonsurgical Orthodontic Camouflage of Adult Skeletal Class III Malocclusion With Anterior and Posterior Crossbites and Pre-existing Alveolar Bone Loss: A Case Report

**DOI:** 10.7759/cureus.116610

**Published:** 2026-09-20

**Authors:** Bolormaa Sainbayar, Khongorzul Myagmarjav, Nursabi Khizatkhan, Dariimaa Ganbat

**Affiliations:** 1 Department of Orthodontics, School of Dentistry, Mongolian National University of Medical Sciences, Ulaanbaatar, MNG; 2 Graduate School of Medical Science, Mongolian National University of Medical Sciences, Ulaanbaatar, MNG

**Keywords:** adult orthodontics, alveolar bone loss, crossbite, dentoalveolar compensation, orthodontic camouflage, premolar extraction, skeletal class iii malocclusion

## Abstract

Adult skeletal Class III malocclusion in non-growing patients may be managed with combined orthodontic-orthognathic surgery or, in carefully selected mild discrepancies, orthodontic dentoalveolar camouflage. We report a 33-year-old Mongolian woman evaluated at the first documented presentation (Day 0) with maxillary anterior and posterior crowding, an anterior crossbite, a left posterior crossbite, and a mild skeletal Class III intermaxillary relationship. Pretreatment cephalometry showed sella-nasion-A point angle (SNA) 74.3°, sella-nasion-B point angle (SNB) 76.0°, and A point-nasion-B point angle (ANB) -1.7°, a pattern indicating predominant maxillary retrusion relative to the mandible. Pretreatment records also demonstrated alveolar bone loss. Treatment used a 0.022-inch high-torque self-ligating fixed appliance, extraction of the maxillary first premolars (Fédération Dentaire Internationale (FDI) 14 and 24), and sequential copper-nickel-titanium, stainless-steel, and titanium-molybdenum alloy archwires, with elastic-chain mechanics during alignment. Active treatment lasted 17 months. The anterior and left posterior crossbites were corrected, maxillary crowding was resolved, dental midlines were aligned, and bilateral Angle Class I canine relationships were achieved. Changes were predominantly dentoalveolar: maxillary central incisor long axis to Frankfort horizontal angle (U1-FH) increased from 103.0° to 108.7° (+5.7°) and maxillary central incisor to nasion-pogonion line (U1-NPog) from 2.8 to 6.1 mm (+3.3 mm), whereas ANB changed minimally from -1.7° to -1.3°. Available records showed stable periodontal supporting tissues, no progression of alveolar bone loss, and no clinically apparent radiographic root resorption. This case illustrates the feasibility of carefully planned nonsurgical camouflage in a selected adult with mild skeletal Class III discrepancy and reduced periodontal support, without establishing the superiority of the self-ligating appliance.

## Introduction

Skeletal Class III malocclusion describes an anteroposterior jaw-base relationship in which the mandible is positioned relatively anterior to the maxilla. The pattern may reflect maxillary retrusion, mandibular prognathism, or a combination of both, and it may be accompanied by dentoalveolar compensation, anterior or posterior crossbite, and an unfavorable facial profile. In non-growing patients, treatment selection commonly lies between orthodontic camouflage and combined orthodontic-orthognathic surgical correction. Orthodontic camouflage seeks to improve the dental and occlusal expression of the discrepancy through controlled tooth movement without repositioning the jaw bases, whereas orthognathic surgery directly changes the skeletal relationship. The choice depends on the magnitude and pattern of the discrepancy, existing dentoalveolar compensation, periodontal support, soft-tissue considerations, and patient-centered factors [1-3].

Evidence supports camouflage in selected adults with mild-to-moderate Class III discrepancies that remain within acceptable dentoalveolar and soft-tissue limits, whereas orthognathic surgery provides greater skeletal correction when the discrepancy exceeds those limits [1-3]. Published case reports also demonstrate that extraction and anchorage strategies are not uniform: they may be tailored to the initial dental compensation, crowding, arch-length deficiency, and treatment objectives [4,5]. Self-ligating bracket systems are widely used fixed appliances. Although reduced ligation friction and other theoretical advantages have been proposed, systematic reviews and meta-analyses do not consistently demonstrate clinically important superiority over conventional brackets in total treatment duration, alignment efficiency, transverse changes, space closure, or most other outcomes [6-9]. Periodontal findings are likewise mixed, with no consistent evidence that bracket design alone determines periodontal health [10,11].

Adults with reduced periodontal support require particularly cautious treatment planning because alveolar bone loss changes the biomechanical environment in which teeth move. Orthodontic treatment can be feasible in a stable reduced periodontium when inflammation is controlled, forces are appropriately managed, and periodontal monitoring is maintained [12]. The present report describes nonsurgical management of an adult skeletal Class III malocclusion with pre-existing alveolar bone loss and analyzes the predominantly dentoalveolar correction achieved over 17 months.

The specific educational value of this case is the use of bilateral maxillary first-premolar extractions to manage maxillary crowding in a patient whose Class III pattern was dominated by relative maxillary retrusion, yet without net maxillary-incisor retraction at treatment completion. The case therefore highlights the interaction between arch-space management, dentoalveolar compensation, and periodontal boundaries rather than any presumed advantage of the self-ligating appliance.

This case was previously presented in poster form as “Clinical Outcomes of Treating Skeletal Class III Malocclusion Using a Self-Ligating Bracket System” at the 68th Annual Scientific Conference of the Mongolian National University of Medical Sciences, Oral Medicine Branch, Ulaanbaatar, Mongolia, in 2026. The present manuscript expands the clinical documentation and evidence-based interpretation of that presentation.

## Case presentation

Initial examination and diagnosis

**Figure 1:**
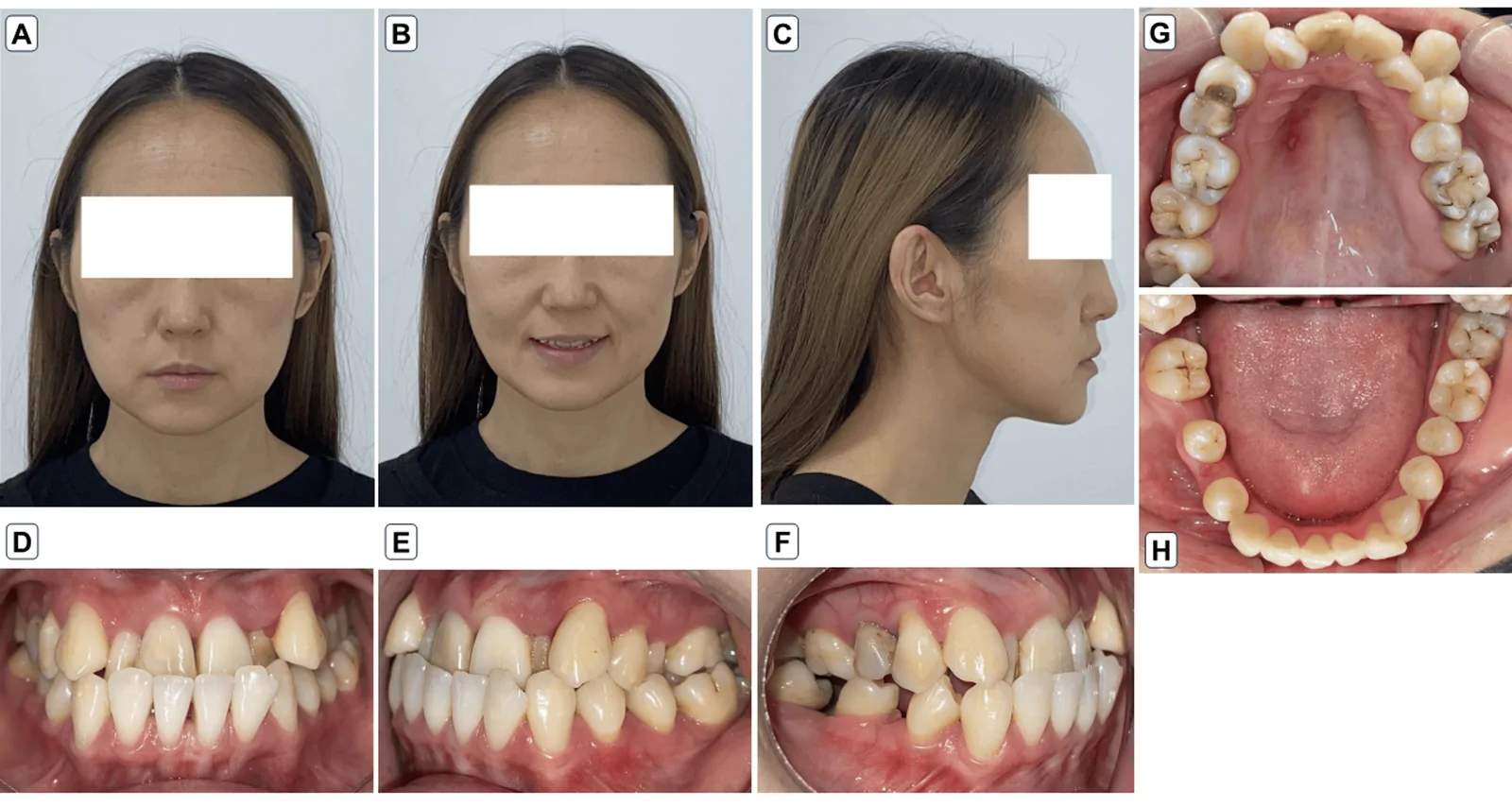
Pretreatment clinical records. Extraoral frontal-at-rest, smiling frontal, and profile photographs (A)-(C) and intraoral frontal, lateral, maxillary occlusal, and mandibular occlusal views (D)-(H) demonstrate the pretreatment Class III facial and occlusal pattern, maxillary anterior and posterior crowding, anterior crossbite, and left posterior crossbite. The periocular regions, including the eyebrows, have been digitally obscured to protect patient identity. Numerical overjet and overbite were not retained in the source documentation and should not be inferred from the photographs.

A 33-year-old woman was evaluated in February 2023 for orthodontic treatment; this first documented orthodontic evaluation is designated Day 0. Pretreatment extraoral records showed a Class III facial pattern in frontal and profile views, while intraoral records documented maxillary anterior and posterior crowding, an anterior crossbite, a left posterior crossbite, and a skeletal Class III malocclusion. The diagnosis was based on lateral cephalometric analysis using digital software, along with digital dental-model measurements. Pretreatment extraoral and intraoral photographs are shown in Figures 1A-1H.

**Figure 2:**
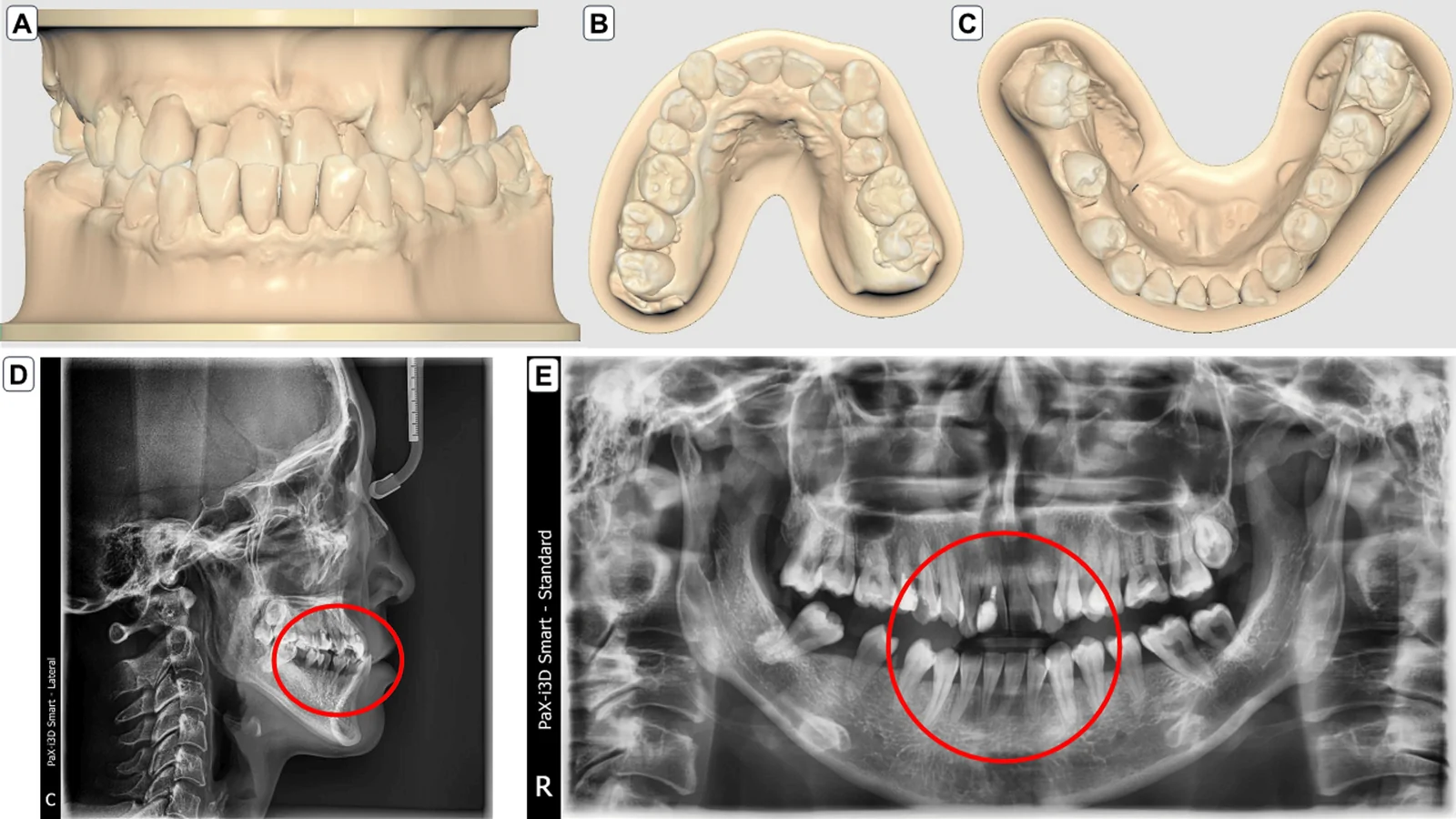
Pretreatment diagnostic records. Digital dental model views (A)-(C) were created using Medit Link, version 3.1.0 (Medit Corp., Seoul, Republic of Korea). The lateral cephalogram (D) and panoramic radiograph (E) document the pretreatment skeletal Class III intermaxillary pattern and reduced periodontal support. Open red circles indicate representative anterior alveolar regions demonstrating the pre-existing alveolar bone loss described in the clinical record; they are illustrative annotations and do not represent standardized quantitative bone-level measurements. Pretreatment cephalometric measurements included SNA 74.3°, SNB 76.0°, and ANB -1.7°. SNA: sella-nasion-A point angle; SNB: sella-nasion-B point angle; ANB: A point-nasion-B point angle.

Complementary pretreatment diagnostic records included digital dental-model views, a lateral cephalometric radiograph, and a panoramic radiograph. The radiographic records demonstrated reduced periodontal support with pre-existing alveolar bone loss, particularly in the representative anterior regions identified in Figures 2A-2E. The retained records did not include standardized quantitative bone-level measurements; therefore, the severity of alveolar bone loss is not expressed retrospectively in millimeters, as a percentage of root length, or as a formal periodontal stage/grade.

A complete periodontal chart was not available in the retained source documentation. Consequently, pretreatment and serial probing depths, bleeding on probing, clinical attachment levels, and standardized mobility scores cannot be reported. Numerical overjet and overbite measurements were also not retained. These values were not estimated retrospectively from photographs or radiographs because doing so would introduce unsupported precision. The report therefore distinguishes directly measured cephalometric changes from qualitative clinical and radiographic observations.

Cephalometric evaluation

Pretreatment cephalometry showed a negative ANB angle of -1.7°, with SNA 74.3° and SNB 76.0°. The values were below the reference ranges used in the original clinical analysis, with a substantially greater deviation of SNA than SNB. Published cephalometric norms for Mongolian adults with normal occlusion similarly place mean SNA near 82° and mean SNB near 79°, supporting the interpretation of this patient's Class III relationship as predominantly maxillary retrusion relative to the mandible rather than absolute mandibular prognathism [13]. The gonial angle was increased at 133.4°, indicating a hyperdivergent tendency. Dentally, the maxillary central incisor was retroclined and retruded relative to the stated reference values (maxillary central incisor long axis to Frankfort horizontal angle (U1-FH) 103.0°; maxillary central incisor to nasion-pogonion line (U1-NPog) 2.8 mm), while the mandibular incisor inclination and position were close to the stated reference ranges (Table 1).

**Table 1:** Cephalometric measurements before and after treatment. Values are reported to one decimal place. Change was calculated as post-treatment minus pretreatment. Reference values are those used in the original clinical cephalometric analysis. These are descriptive paired observations from one patient; inferential statistics, P values, confidence intervals, and measures of heterogeneity are not applicable. SN-FH, sella-nasion to Frankfort horizontal angle; SNA, sella-nasion-A point angle; SNB, sella-nasion-B point angle; ANB, A point-nasion-B point angle; SND, sella-nasion-D point angle; U1-FH, maxillary central incisor long axis to Frankfort horizontal angle; U1-NPog, maxillary central incisor to nasion-pogonion line; L1-FH, mandibular central incisor long axis to Frankfort horizontal angle; L1-NPog, mandibular central incisor to nasion-pogonion line; U1-L1, interincisal angle.

Parameter	Reference	Pretreatment	Post-treatment	Change	Interpretation
SN-FH (°)	7	9.0	9.0	0.0	Stable
SNA (°)	82 ± 2	74.3	74.5	+0.2	Maxillary retrusion; stable
SNB (°)	80 ± 2	76.0	75.8	-0.2	Below reference; stable
ANB (°)	2 ± 2	-1.7	-1.3	+0.4	Mild Class III; minimal change
SND (°)	76 ± 2	74.3	74.2	-0.1	Stable
Gonial angle (°)	119 ± 6	133.4	133.2	-0.2	Hyperdivergent; stable
U1-FH (°)	112 ± 4	103.0	108.7	+5.7	Maxillary incisor proclination
U1-NPog (mm)	9 ± 2	2.8	6.1	+3.3	Forward maxillary incisor movement
L1-FH (°)	63 ± 5	63.7	62.2	-1.5	Within stated range; small change
L1-NPog (mm)	5 ± 2	4.9	4.9	0.0	Essentially unchanged
U1-L1 (°)	131 ± 3	135.8	132.7	-3.1	Toward stated reference

Treatment objectives and progress

Treatment objectives were to resolve maxillary crowding, correct the anterior and left posterior crossbites, improve dental midline relationships, establish a stable functional occlusion, and preserve the periodontal supporting tissues. The available documentation did not contain a formal patient-specific decision analysis between orthognathic surgery and orthodontic camouflage; therefore, this report does not retrospectively state that surgery was declined, contraindicated, or formally rejected.

Orthodontic treatment began on Day 0 using a high-torque 0.022-inch self-ligating fixed appliance. Maxillary first premolars Fédération Dentaire Internationale (FDI) 14 and 24 were extracted for relief of maxillary crowding and space management. Initial 0.014-inch copper-nickel-titanium (CuNiTi) round archwires were placed in both arches. At approximately Day 60, treatment progressed to rectangular CuNiTi archwires, and an elastic chain was used for canine alignment. Subsequent stainless-steel archwires and titanium-molybdenum alloy (TMA) archwires were used for midline correction, occlusal detailing, and finishing. The exact rectangular CuNiTi dimensions, measured force levels, and detailed anchorage data were not clearly documented and therefore cannot be reconstructed (Table 2).

**Table 2:** Documented clinical timeline and orthodontic treatment sequence. Timing is reported only to the level preserved in the retained clinical records. The exact rectangular CuNiTi dimensions, measured force levels, and detailed anchorage values could not be verified and are intentionally not inferred. FDI: Fédération Dentaire Internationale tooth-numbering system; CuNiTi: copper-nickel-titanium; TMA: titanium-molybdenum alloy.

Stage/timing	Documented appliance or event	Documented purpose/outcome
Day 0 (February 2023)	Extraction of FDI 14 and 24; 0.014-inch round CuNiTi in both arches	Initial alignment, relief of maxillary crowding, and space management
Approximately Day 60	Rectangular CuNiTi plus elastic chain	Canine alignment and continued space management
Later treatment	Stainless-steel archwires	Midline correction and occlusal detailing
Finishing	TMA archwires	Final root and occlusal adjustment
Month 17	Completion of active treatment	Anterior and left posterior crossbites corrected; maxillary crowding resolved; dental midlines aligned; bilateral angle Class I canine relationships achieved

**Figure 3:**
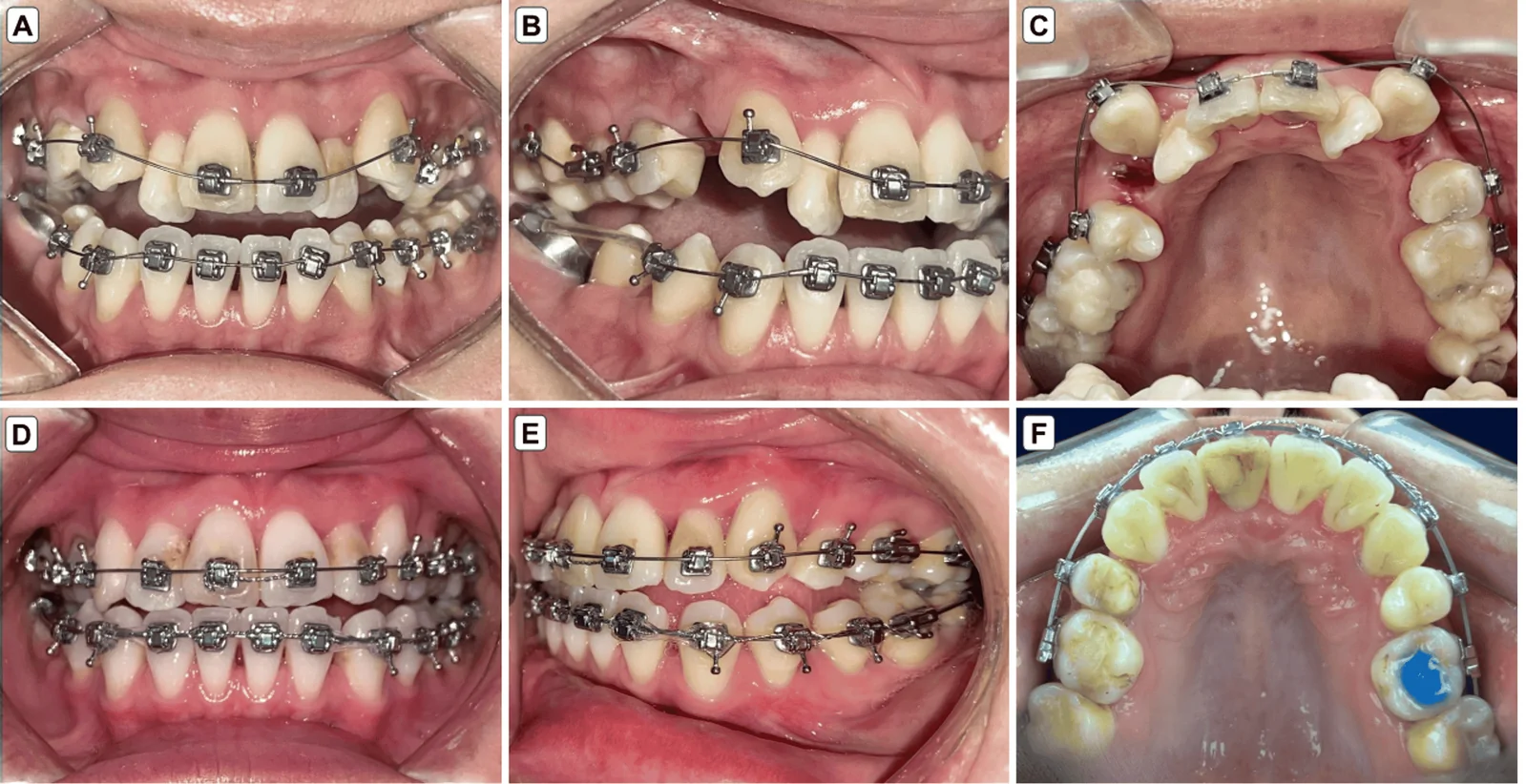
Orthodontic treatment progression. Representative intraoral photographs demonstrate sequential orthodontic treatment. Early records (A)-(C) show initial alignment with a self-ligating fixed appliance and round copper-nickel-titanium (CuNiTi) archwires. Later records (D)-(F) demonstrate progressive alignment and space management, including the phase in which elastic-chain mechanics and subsequent working/finishing archwires were used. The images are representative of treatment progression; the exact rectangular CuNiTi dimensions and applied force levels cannot be determined from the photographs and are not inferred.

Representative intraoral photographs illustrating the corresponding orthodontic treatment progression are shown in Figures 3A-3F.

Treatment outcome

Active treatment lasted 17 months. At completion, bilateral Angle Class I canine relationships were achieved, the anterior and left posterior crossbites were eliminated, maxillary crowding was resolved, the maxillary arch became more rounded, and the dental midlines were aligned. The available records documented correction of the transverse and anteroposterior occlusal relationships but did not provide numerical overjet or overbite measurements.

As a single-patient case report, treatment changes are reported descriptively rather than with inferential statistics. Cephalometric changes were predominantly dentoalveolar. U1-FH increased from 103.0° to 108.7° (+5.7°), and U1-NPog increased from 2.8 to 6.1 mm (+3.3 mm), consistent with maxillary incisor proclination and forward movement. Mandibular incisor position changed minimally, while the skeletal relationship remained essentially unchanged (ANB -1.7° to -1.3°). These findings support a camouflage mechanism rather than skeletal correction.

The retained clinical record described decreased tooth mobility, and the available qualitative clinical and radiographic documentation did not show obvious progression of the pre-existing alveolar bone loss during active treatment. No clinically apparent radiographic root resorption was identified at treatment completion. These observations are clinically reassuring but should not be interpreted as quantified periodontal or radiographic treatment effects because serial probing depths, bleeding scores, clinical attachment levels, standardized mobility measurements, quantitative bone-level measurements, and standardized root-length analysis were unavailable.

Improvement in facial appearance, mastication, and speech was also documented in the clinical record. Because standardized patient-reported outcome measures were unavailable, these observations are reported descriptively rather than as measured treatment effects.

**Figure 4:**
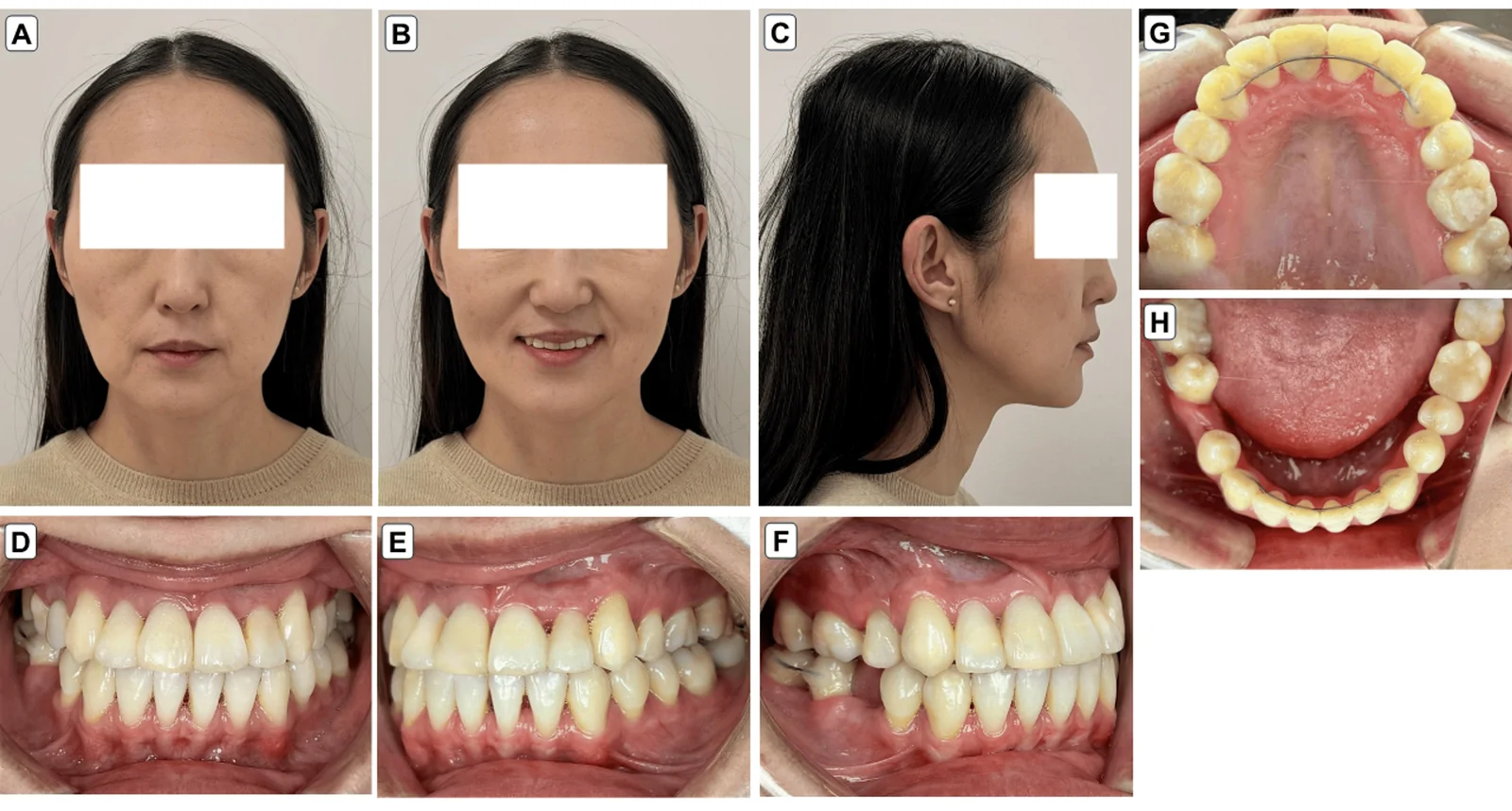
Post-treatment clinical records after 17 months of active treatment. Extraoral frontal-at-rest, smiling frontal, and profile photographs (A)-(C) and intraoral frontal, lateral, maxillary occlusal, and mandibular occlusal views (D)-(H) demonstrate correction of the anterior and left posterior crossbites, resolution of maxillary crowding, improved arch form, aligned dental midlines, and bilateral Angle Class I canine relationships. The periocular regions, including the eyebrows, have been digitally obscured to protect patient identity. These photographs document the clinical appearance at completion of active treatment but do not substitute for quantitative periodontal measurements or long-term follow-up.

Retention and post-debond follow-up were not documented in the available records. Consequently, this report describes the results at completion of active treatment and cannot establish long-term occlusal or periodontal stability. Post-treatment extraoral and intraoral clinical records are shown in Figures 4A-4H.

## Discussion

Clinical decision-making in adult skeletal Class III malocclusion

Adult skeletal Class III treatment requires a clear distinction between correction of the skeletal discrepancy and camouflage of its dental expression. Evidence supports orthognathic surgery when substantial skeletal correction is required, whereas camouflage may be reasonable for selected mild-to-moderate discrepancies within acceptable dentoalveolar and soft-tissue limits [1-3]. In this patient, the pretreatment ANB angle was modestly negative at -1.7°, and post-treatment skeletal measurements changed very little, which is consistent with the expected biological limits of nonsurgical camouflage.

The available documentation did not include a formal patient-specific decision analysis between surgery and camouflage. This report therefore avoids retrospective reconstruction of an undocumented surgical discussion. The clinical value of the case lies instead in the observed dentoalveolar response, the management of maxillary crowding despite relative maxillary retrusion, and the need to work within reduced periodontal support.

Rationale for the maxillary extraction pattern

The extraction choice warrants explanation because removing maxillary premolars in a patient with relative maxillary retrusion can appear counterintuitive. The documented indication for extraction of FDI 14 and 24 was relief of maxillary crowding. Importantly, the post-treatment records do not show net retraction of the maxillary incisors: U1-FH increased by 5.7° and U1-NPog advanced by 3.3 mm. Thus, despite premolar extraction, the anterior segment moved forward overall. This pattern suggests that extraction space was principally used to relieve crowding and coordinate the arch rather than to retract the maxillary anterior teeth.

The mandibular incisor position remained essentially unchanged, so correction of the anterior crossbite occurred predominantly through maxillary dentoalveolar compensation. Published camouflage reports show that extraction and anchorage strategies can vary based on pre-existing dentoalveolar compensation and arch-space requirements [3-5]. The present case therefore highlights the importance of treating the actual space problem rather than assuming that every Class III patient requires the same extraction pattern.

The documented archwire sequence progressed from round CuNiTi to rectangular CuNiTi, stainless steel, and TMA. In a patient with reduced periodontal support, a staged sequence that begins with flexible wires and proceeds to stiffer working wires and controlled finishing mechanics is biologically plausible; however, measured orthodontic force levels, detailed anchorage data, and the exact rectangular working-wire dimension were unavailable. These variables therefore cannot be presented as quantified mechanisms of success, and no retrospective estimates were introduced.

Self-ligating brackets: Interpretation of the evidence

The self-ligating appliance was one component of treatment, but current evidence does not justify attributing the outcome specifically to bracket design. Meta-analyses and systematic reviews have found little or no clinically important advantage of self-ligating over conventional brackets for total treatment duration, alignment efficiency, transverse changes, or space closure [6-9]. Periodontal outcomes are also inconsistent, and bracket type should not be used as a surrogate for periodontal risk control [10,11]. The favorable result is better interpreted as the combined effect of case selection, extraction-based space management, sequential mechanics, clinical execution, and patient-specific biological response.

Periodontal and root-resorption considerations

Orthodontic treatment in adults with reduced periodontal support can be feasible when periodontal inflammation is controlled, and biomechanics are adapted to the reduced supporting tissues [12]. In this case, the retained clinical record described decreased tooth mobility, and the available qualitative clinical and radiographic documentation did not show obvious progression of the pre-existing alveolar bone loss during the 17-month active treatment. These observations are clinically reassuring but do not establish periodontal stability because serial probing depths, bleeding scores, clinical attachment levels, standardized mobility scores, and quantified radiographic bone levels were unavailable. The wording “no obvious progression” is therefore used deliberately instead of implying a measured periodontal treatment effect.

The direction of maxillary incisor movement also deserves attention. Proclination and approximately 3.3 mm of forward movement in a periodontium with pre-existing bone loss may approach the labial alveolar boundary. The available documentation did not include three-dimensional measurements of labial alveolar bone thickness; therefore, the qualitative absence of obvious radiographic worsening should not be interpreted as proof that the labial cortical plate was unchanged. In adults with reduced support, planned tooth movement should remain within the individual alveolar envelope.

External apical root resorption is a recognized adverse effect of fixed-appliance orthodontics and is influenced by multiple patient- and treatment-related factors [14]. The available records showed no clinically apparent radiographic root resorption at treatment completion. Because no standardized quantitative root-length analysis was available, the appropriate interpretation is that no clinically apparent radiographic resorption was identified, rather than that microscopic or small quantitative changes were excluded.

Comparison with published case literature

Published Class III camouflage case reports illustrate the heterogeneity of nonsurgical strategies. Yashima et al. used temporary anchorage devices in a complex Class III open-bite case with ankylosis, whereas Aulia et al. reported fixed-appliance camouflage with Class III elastics in an adult with anterior crossbite and functional shift [4,5]. The present case differs by using bilateral maxillary first-premolar extraction with predominantly maxillary dentoalveolar compensation in a patient with pre-existing alveolar bone loss. The educational point is not the bracket type itself, but the interaction between arch-space management, incisor compensation, and periodontal boundaries.

Limitations

This report describes a single patient and cannot establish comparative effectiveness, periodontal safety, or superiority of any appliance system. It is also constrained by the completeness of the retained clinical record. Quantitative pretreatment and post-treatment periodontal charting, measured radiographic bone-loss values, numerical overjet and overbite measurements, standardized patient-reported outcome scores, detailed anchorage and force data, and three-dimensional assessment of the labial alveolar bone envelope were unavailable. The exact rectangular CuNiTi dimensions could not be verified. The patient-specific rationale for choosing camouflage rather than orthognathic surgery was not documented in the retained records, and retention and post-debond follow-up data were unavailable. Because these missing elements cannot be reliably reconstructed after the fact, the manuscript intentionally avoids estimated values or retrospective assumptions. These limitations restrict causal interpretation and prevent assessment of long-term occlusal or periodontal stability.

## Conclusions

Individualized nonsurgical dentoalveolar camouflage produced the documented occlusal correction in this selected adult with a mild skeletal Class III relationship, maxillary crowding, anterior and left posterior crossbites, and pre-existing alveolar bone loss. Over 17 months, correction was predominantly maxillary dentoalveolar, with substantial maxillary incisor proclination and forward movement and minimal skeletal change. The retained qualitative clinical and radiographic records did not show obvious progression of the pre-existing alveolar bone loss and did not identify clinically apparent radiographic root resorption; however, standardized quantitative periodontal, bone-level, and root-length measurements were unavailable, so periodontal stability or safety cannot be established from this single case. The maxillary premolar extraction pattern illustrates that arch-length deficiency can be managed without necessarily producing net maxillary incisor retraction. The findings should not be interpreted as evidence of the superiority of self-ligating brackets or of long-term stability. Individualized diagnosis, respect for periodontal boundaries, and documented long-term follow-up remain essential.
